# An Overview of Strategies to Improve Vaccination Compliance before and during the COVID-19 Pandemic

**DOI:** 10.3390/ijerph191711044

**Published:** 2022-09-03

**Authors:** Lorena Charrier, Jacopo Garlasco, Robin Thomas, Paolo Gardois, Marco Bo, Carla Maria Zotti

**Affiliations:** 1 Department of Public Health Sciences and Paediatrics, University of Turin, 5/bis Via Santena, I-10126 Turin, Italy; 2 Northern Metropolitan Department Direction, Local Health Authority Turin 3 (ASL TO3), 152 Via Don Giovanni Sapino, I-10078 Venaria Reale, Italy; 3 Biblioteca Federata di Medicina “Ferdinando Rossi”, University of Turin, 5/bis Via Santena, I-10126 Turin, Italy; 4 Hospital Medical Direction, Local Health Authority Turin 5 (ASL TO5), 1 Piazza Silvio Pellico, I-10023 Chieri, Italy

**Keywords:** vaccination policies, childhood vaccines, mandatory, measles, COVID-19

## Abstract

The debate on vaccination mandate was fuelled over the past two years by the COVID-19 pandemic. This study aimed at overviewing vaccination strategies and corresponding vaccine coverages for childhood vaccinations before the pandemic and for SARS-CoV-2 in high-income countries. A qualitative comparison was also performed between the two contexts: unlike for childhood vaccinations, only one European country (Austria) imposed generalised COVID-19 mandates, most countries preferring targeted mandates for higher-risk categories (Italy, Greece) or workers in key public services (Finland, Australia, New Zealand, UK, Germany). Many countries (Norway, Sweden, Netherlands, Portugal, Spain) confirmed their traditional voluntary vaccination approach also for COVID-19, while others (Slovenia and Hungary), historically relying on compulsory vaccination strategies, surprisingly opted for voluntary SARS-CoV-2 vaccination, with unsatisfactory results in terms of immunisation rates. However, no tangible relationship was generally found between vaccination policies and immunisation coverages: data show that, unlike some countries with mandates, countries where vaccinations are merely recommended could achieve higher coverages, even beyond the recommended 95% threshold. The COVID-19 experience has enriched pre-existent vaccination strategy debates by adding interesting elements concerning attitudes towards vaccines in a novel and unexplored context. Interpreting the available results by considering the different cultural contexts and vaccine hesitancy determinants can help to better understand the complexity of the relationship between policies and achieved coverages.

## 1. Introduction

Current vaccination strategies are informed by lessons learned from suboptimal vaccine coverage and infectious disease outbreaks that occurred throughout the European Union (EU) between 2016 and 2018. In response, many countries began imposing stricter rules regulating childhood vaccination, some with compulsory vaccination for certain infectious diseases [1,2,3,4,5,6,7]. According to the World Health Organization (WHO) European Region report, measles outbreaks between 2016 and 2018 involved a total of 83,540 cases and 74 related deaths in 2018, up sharply from 25,869 cases and 42 deaths in 2017, and 5273 cases and 13 deaths in 2016. In 2018 alone, eight countries (Ukraine, Serbia, Israel, France, Italy, Russian Federation, Georgia, Greece) reported over 2000 cases each. Between December 2018 and November 2019, the highest number was recorded for France (2674 cases, 20%), followed by Romania (1746, 13%), Italy (1689 cases, 12%), Poland (1532 cases, 11%), and Bulgaria (1201, 9%). No country reported no cases [8]. Based on 2017 data, measles was eliminated in 37 of the 53 countries in the WHO European Region, while it was still endemic in Belgium, France, Germany, Italy, Romania, and the Russian Federation according to the European Regional Verification Commission for Measles and Rubella Elimination (RVC) [9].

Outside Europe, in 2019 alone, about 1300 measles cases were reported in the United States, the highest since 1992, although measles was declared eliminated in 2000. The 2019 outbreaks were linked to travel-related cases that reached a population of un- or under-vaccinated people [10]. 

The European Vaccine Action Plan 2015–2020 (EVAP), unanimously adopted by the 53 countries of the WHO European Region, states that at least 95% of individuals in a population must be immune to ensure community protection for everyone—including children too young to be vaccinated and individuals ineligible for immunisation due to comorbidities and medical conditions. While the 95% coverage rate is necessary only for diseases with a higher R_0_ (measles, chicken pox, whooping cough), for practical reasons this target has been set for all vaccinations against infectious diseases [11].

Attempts to reach (and maintain) this ambitious goal had chequered success, however. Immunisation measures and strategies range widely from voluntary choice-oriented policies to penalties, financial incentives, and social restrictions (the unvaccinated barred from attending school), to mandatory regulations [1,2,3,4,5]. The ongoing debate on whether to make vaccination mandatory was fuelled over the past two years by the SARS-CoV-2 (COVID-19) pandemic, which has taken public health experts by surprise due to its infectious potential and rapid spread worldwide [12]. 

The availability of safe and effective vaccines that can prevent most infections and reduce the severity of the disease has once again raised the importance of successful strategies to achieve vaccine coverage objectives, and highlighted the conspicuous differences in immunisation policies, vaccine hesitancy, and vaccination coverage among countries. Furthermore, disruption of vaccination services during the COVID-19 pandemic implied that millions of children missed their routine childhood vaccine doses, setting the stage for future outbreaks without the coverage to prevent them [13,14,15,16].

The study aims to provide an overview of strategies to promote childhood vaccination adherence in high-income countries. Data on vaccination coverage from 2015 and 2019 are compared to reveal changes before measles outbreaks and after the implementation of new or stricter vaccination policies and test their relationship to coverage. Strategies for COVID-19 vaccination and vaccination coverage in the last 12 months are also explored, and issues potentially related to vaccine hesitancy are discussed.

## 2. Materials and Methods

The data reported in the manuscript refer to a set of countries considered representative of different vaccination strategies: “mandatory”, “voluntary with requirements”, “voluntary with financial incentives”, or “totally voluntary”. This set included some countries that recently had to deal with measles outbreaks, along with some other (geographically and sometimes culturally close to the former) that managed to prevent the problem with strategies to maintain high vaccination coverage for childhood diseases, and some countries that, albeit geographically distant (US, Australia), have adopted policies that, if effective, could be used in countries not attaining the recommended coverage yet. The same countries were studied in relation to the vaccination strategy deployed to tackle the pandemic, and to the corresponding results in terms of vaccination coverage.

Measles was the focus of our attention in the pre-pandemic analysis for three reasons: first, before the COVID-19 pandemic, measles was the most recent epidemic reported by many countries that dealt with the event often via different approaches; second, eliminating measles in five of the six WHO regions by 2020 was the goal of the 2012 Global Vaccine Action Plan endorsed by the World Health Assembly [17] (despite the long history of MMR vaccination with a safe and effective vaccine, the elimination of measles remains an unfulfilled goal that still requires effective public health and policy efforts); finally, immunisation Agenda 2021–2030 uses measles incidence as a quality indicator of public health systems and the strength of immunisation systems [18].

Data about measles vaccination coverage were downloaded from the World Health Organisation (WHO), United Nations International Children’s Emergency Fund (UNICEF), Centers for Disease Control and Prevention (CDC), and the World Bank databases [19,20,21,22,23].

Years 2015 and 2019 were chosen as representatives of childhood vaccination coverage before and after the 2016–2018 infectious disease revival: 2015 as it was the last year preceding the measles epidemic waves in Europe and the following restrictions adopted in some countries; 2019 as it could at least partially reflect the effects of the mentioned new/stricter regulations and because it was the last year with expectedly reliable data about childhood vaccine coverages before the COVID-19 pandemic (in fact, 2020 data might have been blurred because of the pandemic-related disruption in data collection and non-COVID vaccination campaigns).

Data on COVID-19 vaccination coverage were obtained from the “Our World in Data” COVID vaccination database, to have up-to-date and comparable data between countries [24]. 

A narrative review of the literature was carried out on a wide variety of data sources for vaccination policies. Information on childhood and COVID-19 immunisation programs was obtained from the European Centre for Disease Prevention and Control (ECDC) [25,26] and from the institutional websites of the countries included in the study (ministries of health and other regulatory agencies). We searched press agency and newspaper websites (Reuters, ANSA, The Guardian, CNN, France24) to identify strategy changes regarding compulsory COVID-19 vaccination. 

We referred to Haverkate et al. [27] and Attwell et al. [4] for the definition of “mandatory” and “recommended” or “voluntary” vaccination.

## 3. Results

### 3.1. Childhood Vaccination Policies: Measles

According to the ECDC, measles vaccination is now mandatory for children aged 11–23 months in 10 out of 30 member states (Bulgaria, Croatia, Czech Republic, France, Hungary, Italy, Latvia, Poland, Slovakia, Slovenia) [25]. The concept of “mandatory vaccination” varies widely, ranging from legally required albeit mildly enforced, to fines imposed on non-compliant citizens or parents responsible for having their children vaccinated, social restrictions, financial penalties, or incentives [1,4,6].

The strictest regulations regarding vaccination are enforced in Slovenia, Italy, and France. Slovenia has one of the most rigorous vaccination policies based on the principle that the benefits of vaccination for the health of an individual and the wider community outweigh any possible harm that may occur to the individual due to vaccine-related adverse effects [28]. Since 1995, vaccination against nine diseases (diphtheria, tetanus, poliomyelitis, pertussis, *Haemophilus influenzae* type b, measles, mumps, and rubella for infants, and hepatitis B before starting school) is mandatory for participation in social activities [29]. 

In Italy, vaccination against four diseases (diphtheria, poliomyelitis, tetanus, hepatitis B) was mandatory under the former schedule and was required (until 1999) to be admitted to school, while other vaccines, including that against measles, were “recommended”. Due to a decline in immunisation levels below the coverage targets set by Italian [30] and international standards [11], in 2017 a new decree (Law 2017/119) introduced mandatory vaccination against six more diseases (pertussis, *Haemophilus influenzae* B, chickenpox, measles, mumps, and rubella) [31]. This change aroused social and political debate between those supporting mandatory vaccination, because they were worried about the 2017 measles outbreak, and those advocating the principles of self-determination in health-related choices [32].

A similar change in the vaccination schedule was made in France. Before 2017, vaccination was mandatory against three diseases (diphtheria, tetanus, poliomyelitis) and recommended against eight others. Although vaccination was mandatory for admission to schools, kindergartens, day-care centres, and summer camps, the regulation was seldom enforced (fines and imprisonment). As vaccination coverage remained high for the mandatory and the recommended vaccines combined with them, but lower for vaccines that were only recommended, vaccination mandates were extended from 3 to 11 for children born 1 January 2018 onwards (Law 2017/1836) to include pertussis, *Haemophilus influenzae* B, hepatitis B, pneumococcus, meningococcus C, and the measles, mumps, and rubella (MMR) complex [4,33]. In Italy and France, non-vaccinated children are barred from entering a public educational institution (e.g., kindergarten, schools, or preschools); exemption is granted on proof of medical contraindication to vaccination.

In Germany, where all vaccines had been voluntary though strongly recommended [4], the 2017 amendment to the Infection Protection Act (IfSG) [34] mandated kindergartens to notify health agencies when parents refused to provide proof of their child’s vaccination, so that the health department could invite the child’s parents or legal guardian for a consultation or fine them. The 2019 Measles Protection Act (*Masernschutzgesetz*) [35] made measles vaccination compulsory for children as of March 2020 under penalty of a fine or a ban from attending nursery or primary school. The law extended mandatory vaccination to specific professional figures, such as holiday camp educators, teachers, professors, and healthcare workers.

Other countries rely on individual responsibility, with more or less strict limitations. In Australia, for example, all vaccinations are voluntary; however, a system of state incentives and disincentives is in place to ensure that high immunisation rates can be maintained: only parents of vaccinated children are eligible for family tax benefits or child care fee reductions (“No Jab, No Pay” policy) [36]. In some states (e.g., New South Wales, Queensland, and Victoria), children must be fully immunised to attend childcare services (“No Jab, No Play” policy) [37]. Exceptions are granted only to persons with a documented history of anaphylaxis or immunocompromised state [4]. 

In the United States, 49 states and the District of Columbia require the MMR (measles, mumps, rubella) vaccine for kindergarten entry and attending school. Iowa is the only state that requires the measles and rubella vaccine but not the mumps vaccine [38]. However, exemptions–varying from state to state–were formerly granted not only for medical reasons, but also for religious objections (in 44 states and the District of Columbia) or personal, moral, or other beliefs (in 15 states), with no substantial differences in vaccination coverages, ranging from 88% to 94% in all exemption categories [22,23,38]. With the view to increase coverage rates, recent immunisation legislation eliminated personal belief exemptions for school entry, added or strengthened vaccination requirements, and promoted vaccine education programs [39]. 

In Canada, where the national constitution prohibits the enactment of legislation that directly mandates actions to enforce vaccination (and, therefore, legislation is mandated by provincial acts), measles vaccination is compulsory when registering children for school in three provinces (Ontario, Manitoba, New Brunswick). An exemption may be granted for medical, religious, or conscientious reasons [40,41].

Most European countries (the United Kingdom, The Netherlands, Portugal, Spain, Greece, and the Scandinavian countries) offer vaccines on a voluntary basis. Vaccination is strongly recommended; children are vaccinated before registration for childcare centres or school, but an immunisation certificate is not required for attending school. Adherence to immunisation programmes relies on individual responsibility and trust in vaccines, as well as vaccination education and financial support. In the UK and Finland, vaccinating doctors receive a refund for each person they vaccinate [42]. The Netherlands maintains a central digital vaccination registry that holds individual-level data and a call–recall system that reminds eligible citizens to get vaccinated, thus, ensuring a high level of coverage [3]. This is not an isolated instance, as many countries that opt for a voluntary approach often rely on a system of reminders that provide information about vaccines and notification of due dates either in written form (as, for instance, in Denmark [43]) or through apps or electronic devices, as in New Zealand [44]. Portugal has never mandated compulsory vaccination, except for tetanus and diphtheria in particular cases. Since 2017 [45], Portuguese schools must notify health boards about unvaccinated children. The intent is to promote counselling and education, as well as to raise awareness of the benefits of this public health policy. A similar approach has been adopted by New Zealand, where the National Immunisation Schedule envisages the offer of routine vaccines free of charge to babies, children, and adolescents (including rotavirus and human papillomavirus vaccines), but no compulsory vaccination [44].

### 3.2. COVID-19 Policies

The policy framework for COVID-19 vaccination is highly diverse and evolves with the epidemiological circumstances. This makes it difficult to summarise the strategies implemented. A brief overview of the current situation at the time of writing is given below.

COVID-19 vaccination is generally not mandatory; nevertheless, many countries, including those with a history of voluntary vaccination, have made it compulsory at least temporarily and/or for specific subgroups (e.g., healthcare workers). Although not a legal requirement, a vaccination certificate must be presented to access public venues such as restaurants, museums, and sports facilities [26].

Austria is the only European country that made vaccination compulsory for all nationals 18 years of age or older, whilst in Italy and Greece vaccination is compulsory for specific age groups (above age 50 and 60 years, respectively). This was to protect the population segment at higher risk of COVID-related morbidity, hospitalisation, and mortality. Italy made vaccination mandatory for healthcare workers in April 2021, and then in December 2021, extended mandatory vaccination to school staff and faculty and people working in the police and the armed forces [46,47,48,49].

Similarly, the Czech Republic initially planned for older adults (>60 years) and people in certain professions (e.g., medical personnel, police, firefighters, medical students) to be vaccinated against SARS-CoV-2, but the plan was cancelled by the new government in January 2022 [50]. Germany weighed the opportunity to impose compulsory vaccination for adults aged 18 years or older, but then the idea was reconsidered and, presently, it is unclear whether and when compulsory vaccination will be initiated [51]. 

In France, vaccination is mandatory for healthcare workers since October 2021 [48]. Starting in November 2021, home care workers in the UK (unless exempt) must have received two doses of an approved COVID-19 vaccine. The UK mandate, envisaging the possibility that unvaccinated workers be reallocated to non-frontline tasks or left on unpaid leave until the completion of their two-dose vaccination course, had been planned to be extended to all frontline healthcare and social workers (e.g., porters and receptionists who might have contact with patients) in April 2022, but was revoked on 31 January 2022 [48] ahead of lifting most restrictions between February and March 2022 [52]. 

Outside Europe, Indonesia, Micronesia, Tajikistan, and Turkmenistan imposed COVID-19 vaccine mandates on the adult population [46]. Ecuador also made vaccination mandatory, except for people with medical conditions, while Costa Rica was the first country in the world to require COVID-19 vaccination also for minors: children aged 5 years or older must be vaccinated, except those with a documented medical exemption [53].

In the United States, the Occupational Safety and Health Administration (OSHA) took a federal approach in November 2021, when it released the vaccine-or-test emergency temporary standard (ETS) to protect unvaccinated employees from workplace coronavirus exposure by strongly encouraging they be vaccinated [54]. Under this rule, covered employers are expected to develop, implement, and enforce a mandatory COVID-19 vaccination policy [55]. Nevertheless, after a judicial stay by the Supreme Court, the ETS was withdrawn in January 2022 [56]. Since then, individual state-level approaches have been taken, with some states introducing mandates for employees but others banning or blocking vaccine mandates [48]. Moreover, on 18 August 2021, the White House issued a Fact Sheet announcing that new regulations would be forthcoming, requiring that nursing home workers be fully vaccinated against COVID-19 for the facility to continue receiving Medicare and Medicaid funding [57]. In the meantime, many states have filed legal challenges to the Centres for Medicare and Medicaid Services (CMS) mandate.

Canada has the highest vaccination rates in the world, yet in October 2021 the government opted for even more stringent provisions, by establishing that public sector workers be fully vaccinated to enter the workplace, as well as persons aged 12 years or older to board trains and aeroplanes [58]. Since then, protests erupted across the entire country. Canada Unity, an anti-public-health-mandate group, organised a lorry convoy to Ottawa to protest against the COVID-19 vaccine mandate for truckers crossing the Canada–US border [59]. In February 2022, Canadian Prime Minister Trudeau exceptionally recurred to the powers provided to him by the Emergencies Act to institute further anti-COVID restrictions and halt the transportation worker protests [60].

The Australian Government Department of Health states that “Vaccination for COVID-19 is voluntary—as are all vaccinations in Australia—and people maintain the option to choose” [61]. Nevertheless, since September 2021, proof of vaccination is required for leaving or re-entering the country and for continued employment in areas, such as for workers in old age residential facilities. In February 2022, with the emergency situation improving, Australia loosened its restrictions: as of February 21, all visa holders who are fully vaccinated for international travel purposes can travel to Australia. Australian citizens and permanent residents aged 12 years or older who are fully vaccinated can leave Australia without needing an outwards travel exemption and may be eligible for reduced quarantine when returning to Australia [62].

Finally, in New Zealand, COVID-19 vaccination is compulsory for health and disability, education, fire and emergency, police, defence, and correctional workforces, with very few exemptions granted [63]. The mandate is based on the key public service role of these workers, as they work with populations either unable to be vaccinated (education and health) or at increased risk of severe illness from COVID-19 (health and correction), or where outbreaks have occurred overseas (correctional facilities).

### 3.3. Regulation Map and Vaccination Coverage for Measles and COVID-19

Table 1 presents a comparison of measles vaccination coverage in 2015 and 2019 (children aged 12–23 months) in countries deemed representative of different vaccination strategies (“mandatory”, “voluntary with requirements”, “voluntary with financial incentives”, or “totally voluntary”) implemented to reach and maintain recommended coverage. Immunisation strategies and recent COVID-19 vaccination coverage rates are also reported.

The data show that most countries share voluntary vaccination policies for childhood immunisation and that the countries with mandatory vaccination policies do not necessarily achieve higher coverage rates. This is confirmed by the case of the United States, where the recommended 95% threshold has not been achieved yet, although MMR vaccination is required for school entry (despite a wide range of admitted exemptions). Nevertheless, in Italy, where vaccination coverage for MMR was found to be very low (<90%) in 2015, and epidemic outbreaks occurred between 2017 and 2018, vaccination coverage turned out to be 9 percentage points higher two years after vaccination became mandatory [64,65,66]. Although not yet reflected in the data presented in Table 1, vaccination coverage for children born in 2018 also increased notably in France after vaccination became mandatory: the increase in MMR first dose vaccination coverage is around 3% [33]. Conversely, the European countries (Portugal, Spain, Greece, Scandinavian countries) that merely recommend childhood MMR vaccination achieved and were able to maintain vaccination coverages above the recommended 95% threshold. 

As with childhood vaccinations, countries with no legal obligation (e.g., Portugal and Spain) achieved very high COVID-19 vaccination rates. In contrast, Slovenia, which has one of the strictest mandatory childhood vaccination policies in Europe, has maintained COVID-19 vaccination on a voluntary basis and achieved low vaccination coverage.

## 4. Discussion

This overview of public health policies on routine vaccination against childhood diseases, more recently against SARS-CoV-2, and vaccination coverage reveals a highly diversified picture of policies, but no evident link between such policies and outcomes: very high vaccination rates are achieved in countries that rely on voluntary vaccination (e.g., Norway, Finland, Spain, Portugal), while similar or lower rates are reported for countries with stricter regulations (e.g., Italy, France, Slovenia).

This consideration, noted in the literature [2] and confirmed by our summary for measles vaccination, emerged more clearly during the current pandemic. For example, Hungary imposed compulsory vaccination for certain professional categories, yet fell behind Spain or Norway, where coverage of up to 80–88% was attained without COVID-19 vaccination mandates. Nonetheless, almost all countries, including those with recommendation-based vaccination policies, envisaged specific (e.g., Italy) or general (e.g., Austria) vaccination mandates or imposed restrictions on access to public venues (e.g., Greece, the Netherlands), to counteract the spread of the infection [67].

It might be thought that the outbreak of an uncommon and/or severe disease would elicit risk perception and motivate the vaccine hesitant to be vaccinated [68,69]. It might have been expected that the rapid spread of the pandemic would have been a sufficient warning to acquire protection by vaccination, as was partially seen during the measles outbreaks in Europe between 2016 and 2018. Nevertheless, anti-vaccination sentiment and vaccine hesitancy are entrenched attitudes. We believe that our study findings should be read in the framework of vaccine hesitancy and its determinants, in which hesitancy may be defined as a product of vaccine “confidence”, “complacency”, and “convenience” [70]. As public health agencies stepped up their efforts to ensure vaccine *convenience* (i.e., on-site availability and safety) for the public, the two other factors are likely to offer the most plausible explanations for the observed differences in vaccination coverage.

Social and cultural elements exert a strong influence on vaccine *confidence* (i.e., trust in vaccines and vaccination policies) and on a person’s willingness to respond to vaccination campaigns. Even the possibility to introduce and maintain a mandatory vaccination policy depends on the cultural background of a population: during the 19th and the 20th century, mandatory vaccinations were introduced in some eastern and Mediterranean European countries, but such a policy would have never been acceptable in northern European countries [43], neither in the US and in Canada, where the Constitution denies the right of the State to impose such treatments. Not coincidentally, many countries with different policy approaches opted for school mandates, an effective strategy that can make the choice not to vaccinate a child seriously disadvantageous, to increase immunisation rates among children and adolescents [71]: California and Australia restricted exemption to vaccination school mandates to medical contraindication only, Italy and France re-introduced school mandates and fines.

Moreover, immunisation rates are influenced by several other factors, which are independent of the vaccination policy adopted. One predictor of high vaccination compliance is higher education level [72]. Analysis of the country demographics shows that adult education level counts as a possible determinant: the proportion of 55- to 64-year-olds with tertiary education (e.g., Italy, Hungary) is low in the countries with stringent vaccine regulations and/or low coverage rates where such regulations are not in place. By comparison, 30 to 50% of the same age group in the countries with high coverage rates but no or few restrictions (e.g., Norway, Spain, Sweden, Finland) had received tertiary education [73]. Additionally, religious factors may also influence personal beliefs about vaccines [74]. Although religious leaders generally encouraged people to be vaccinated against COVID-19 [75,76], many people view vaccination as conflicting with their religious beliefs, with the subsequent establishment of clusters of unvaccinated in some communities, as occurred in the Netherlands [74] and the United States [77].

The remaining hallmark of vaccine hesitancy (i.e., *complacency*) derives from personal attitudes to vaccines in general: vaccines can be perceived as either a necessary or a useless (potentially harmful) social norm [70]. This touches on the perception of the actual need for prevention. In a given instance, a vaccination campaign may paradoxically become “a victim of its own success” [78], when vaccination might no longer be perceived as necessary because the frequency of an infectious disease is low and because the risks are seen as outweighing the potential benefit to an individual’s health [79,80].

In Italy and France, for example, the relatively low incidence of vaccine-preventable diseases in the last few decades was sufficient reason for many people to opt-out of vaccination, leading to a decline in vaccine coverage. Differently, a striking mismatch can be observed between the low vaccination coverage against COVID-19 (mostly voluntary) and the high rates of childhood vaccination (mandatory) in Slovenia and Hungary, where we might speculate that the vaccination rates result from compliance with legal mandates (an aftereffect of the Soviet system), rather than from actual confidence in science or vaccination programs.

Anti-vax influencers can have a determinant effect on vaccine hesitancy by spreading, via social networks, misinformation about the benefits of vaccination (e.g., minimising the role of vaccination in infection eradication, elimination, and control) and by undermining confidence in vaccination campaigns by spreading fake news about vaccine effectiveness and safety, or exaggerating adverse effects. Furthermore, anti-vax movements have political implications, as shown by Italy in 2017, where many politicians initially appeased their constituents’ demand for voluntary vaccinations, and more recently during the COVID-19 pandemic in Canada, where protests by transport workers on the US–Canadian border led to many restrictions being either lifted or no longer imposed, ensuing in a domino effect in other countries.

On the other hand, a powerful weapon to counteract the harmful effects of anti-vax propaganda is represented by effective, correct, and consistent scientific communication, ideally performed coherently between different information levels and media technologies, which is key to reassuring people about vaccine safety and utility of vaccination campaigns [81]. Of note, the same countries where strong anti-vax movements were present suffered from a lack of communication from healthcare professionals and authorities, and poor coordination among the different institutions involved. Disagreeing opinions were recorded even among physicians and scientists themselves [82,83], which opened the way to uncontrolled (and influential) information spreading through many sources (chiefly social networks [84]) about vaccines.

The present study has both limitations and strengths. Most of the data came from high-income countries, which opens the way to similar studies of different economic contexts for policy comparison; nevertheless, these countries provide reliable (and retrievable) data about public health policies and vaccination coverage. The choice of the countries, in fact, is connected to the limited availability of systematically updated archives for accessing institutional information and international comparisons: information about vaccination policies was retrieved from a variety of sources (e.g., institutions and newspaper websites), which was already crucial for measles, to detect changes in strategies after corresponding outbreaks, and even more in the case of COVID-19, for which vaccination policies changed almost every week in some contexts. This prevented us from including a detailed analysis of countries in Asia or Africa as, for instance, childhood vaccination strategies are nearly unknown in most African countries. However, this choice allows the study to be grounded on thorough research of journal articles, grey literature, online news, and national regulations (often evolving over time and differing from region to region, as in Italy, where the mandate should have standardised the national immunisation program).

## 5. Conclusions

Our study provides a general overview of vaccination strategies against a common childhood disease (measles) and a then unknown disease (COVID-19), and a comparison of vaccination coverage rates in high-income countries. The analysis shows almost no consistency between vaccination policies for COVID-19 and those for routine childhood vaccinations. If some countries have taken a voluntary approach (e.g., Spain, Portugal, Norway) and others mandate vaccination (e.g., Italy) in both contexts, the approach to vaccination against SARS-CoV-2 often differs from that against childhood disease. Several countries with compulsory childhood vaccination opted for voluntary COVID-19 vaccination (e.g., Slovenia, Hungary), often achieving low coverages, while others with recommended schedules for childhood diseases imposed strict mandates for COVID-19 vaccination (e.g., Austria, Canada).

Finally, the link between immunisation policy and actual vaccination coverage is weak. Though some of the variability in policy-driven effects remains unexplained, the determinants of vaccine hesitancy provide insights into the phenomenon. As such, they warrant further study that may inform targeted actions: information that is clear in content and delivery [85], proper training of healthcare operators, medical preparedness, and education about vaccine-related risks [32]. A similar analysis in low- or low-middle-income countries could help to differentiate between various contexts and to better focus on key determinants for building prevention campaigns specific to socioeconomic settings.

## Figures and Tables

**Table 1:** Measles and COVID-19 vaccination strategies, and corresponding vaccination coverages achieved.

Country	Measles				COVID-19	
	Strategy	2015	Trend	2019	Strategy	2022 *
Slovenia	Mandatory	94	=	94	Voluntary Proof of vaccination, recovery, or negative test for employees and access to public venues	59% (61%)
Hungary	Mandatory	99	=	99	Mandatory for healthcare workers, state schoolteachers, and employees at state institutions	64% (66%)
Italy	Mandatory (from 2017)	85	↑↑	94	Mandatory for adults aged >50 years, for all workers, school and university staff, police, and armed forces. Proof of vaccination or recovery to access public venues	78% (84%)
France	Mandatory (from 2018)	91	↓	90	Mandatory for firefighters, healthcare, and transport workers; workers in contact with the public, and foreign athletes Proof of vaccination or recovery to access public venues	77% (80%)
Germany	Mandatory (from 2020)	97	=	97	Mandatory for healthcare workers Planned to make vaccination mandatory for adults	75% (76%)
United States	Voluntary with requirements	92	↓	90	Mandatory for entry into the country	64% (76%)
Australia	Voluntary with financial incentives	95	=	95	Mandatory for healthcare workers in residential nursing homes	79% (85%)
Austria	Voluntary	96	↓	94	Mandatory as of Feb 2022 for adults aged >18 years	72% (75%)
Canada	Voluntary	89	↑	90	Mandatory for federal public service employees	81% (85%)
Finland	Voluntary	95	↑	96	Mandatory for healthcare workers in close contact with elderly or at-risk patients	76% (81%)
Greece	Voluntary	97	=	97	Mandatory for adults aged >60 years and for healthcare workers Proof of vaccination or recovery to access public venues	72% (76%)
Netherlands	Voluntary	95	↓	94	Voluntary Health pass mandatory to access public venues	72% (78%)
New Zealand	Voluntary	93	↓	92	Mandatory for workers in healthcare and disability, education, fire and emergency, police, armed forces, and correctional workforces	77% (83%)
Norway	Voluntary	95	↑	97	Voluntary	73% (79%)
Portugal	Voluntary	98	↑	99	Voluntary COVID certificate mandatory to access public venues Negative test mandatory for entry into the country Negative test to enter residential care homes, healthcare facilities, sports venues, and cultural events, also for the vaccinated	91% (95%)
Spain	Voluntary	96	↑	98	Voluntary	83% (88%)
Sweden	Voluntary	98	↓	97	Voluntary Digital vaccination certificate for indoor public events attended by >100 people, extended to restaurants, cultural venues, and leisure centres (no legal requirement)	74% (77%)
UK	Voluntary	93	↓	91	Mandatory for health and home care workers	72% (77%)

* Data updated to 19 February 2022: % fully vaccinated (% fully + partially vaccinated).

## Data Availability

No new data were created or analysed in this study. Data sharing is not applicable to this article.
